# Migratory destinations and spatial structuring of humpback whales (*Megaptera novaeangliae*) wintering off Nicaragua

**DOI:** 10.1038/s41598-023-41923-7

**Published:** 2023-09-13

**Authors:** Joëlle De Weerdt, Aldo S. Pacheco, John Calambokidis, Melvin Castaneda, Ted Cheeseman, Astrid Frisch-Jordán, Frank Garita Alpízar, Craig Hayslip, Pamela Martínez-Loustalot, Daniel M. Palacios, Ester Quintana-Rizzo, Nicola Ransome, Jorge Urbán Ramírez, Phillip Clapham, Tom Van der Stocken

**Affiliations:** 1 Association ELI-S, Education, Liberté, Indépendance-Scientifique, Allée de Verdalle 39, 33470 Gujan-Mestras, France; 2 Biology Department, Vrije Universiteit Brussel, VUB, Pleinlaan, 1050 Brussel, Belgium; 3 Facultad de Ciencias Biológicas, Universidad Nacional Mayor de San Marcos, Av. Carlos Germán Amezaga #375, Lima, Perú; 4 Cascadia Research Collective, 218½ W 4th Avenue, Olympia, WA 98501 USA; 5 Fundación Naturaleza El Salvador, San Salvador, El Salvador; 6 Happywhale.com, Marine Ecological Research Centre, Southern Cross University, Lismore, NSW Australia; 7 Ecología y Conservación de Ballenas, A.C. ECOBAC, Puerto, Vallarta, México; 8 Department of Fisheries, Wildlife, and Conservation Sciences, Oregon State University, Newport, OR USA; 9 Marine Mammal Institute, Oregon State University, Newport, OR USA; 10 Departamento de Ciencias Marinas y Costeras, Universidad Autónoma de Baja California Sur, La Paz, México; 11 Emmanuel College|Simmons University, Boston, USA; 12 Murdoch University (Harry Butler Institute), Perth, WA Australia; 13 Seastar Scientific, 27605 Hake Rd SW, Vashon, WA 98070 USA

**Keywords:** Animal migration, Conservation biology

## Abstract

Understanding the migratory patterns of large whales is of conservation importance, especially in identifying threats to specific populations. Migration ecology, including migratory destinations, movements and site fidelity for humpback whales (*Megaptera novaeangliae*) remain poorly studied in parts of the range of the Central America population, considered endangered under the United States Endangered Species Act. This study aimed to investigate the migratory destinations of humpback whales sighted at two study sites in Nicaragua, which are part of the Central America population. A ten-year photographic database of humpback whales observed off Nicaragua was combined with citizen science contributions and sightings from dedicated research programs. The resulting image collection was compared with available historical photo identifications and databases using an automated image recognition algorithm. This approach yielded 36 years of photographic identification totaling 431 recaptures in Nicaragua (2006–2008 and 2016–2021) and 2539 recaptures (1986–2020) in both feeding and breeding grounds of 176 unique individuals sighted in Nicaragua. Our results showed that photo-identified whales were recaptured between October and April in breeding grounds and year-round in feeding grounds between British Columbia and California, with peak recaptures between June and October. Our study provided first-time evidence on fine-scale site affinity of individual humpback whales within Nicaraguan waters and to other breeding and feeding grounds.

## Introduction

Animal migration is a common phenomenon among marine taxa^1^. Migratory populations of marine megafauna are often the focus of conservation efforts, particularly in light of increased anthropogenic threats (*e.g.,* vessel collision mortality, fishing gear entanglements) and potential effects of environmental changes^2–4^. Humpback whales (*Megaptera novaeangliae*) migrate between summer feeding grounds at high latitudes and winter breeding grounds at tropical and subtropical latitudes^5,6^. Identifying individual humpbacks from the unique markings and patterns on the ventral surface of their flukes^7,8^ has provided extensive information about the behavior, movements and demographics of this species^9–12^. Migratory patterns of humpback whales can be inferred through collaborative international photographic-identification (hereafter photo-ID) efforts^13,14^ and/or by other techniques such as telemetry (*e.g.,*^15–20^). Understanding migratory movements and site preferences is important to identify site-specific threats such as ship strikes^21^, entanglement in fishing gear^2^ (*e.g*., crab fisheries along the United States (U.S.) West Coast^22^), and disturbance from eco-tourism^23^. Assessment of these threats allows the development of effective international conservation actions for endangered demographic units like the Central America humpback whale population. Population recovery plans for humpback whales and international conservation efforts rely on the type of information provided in this study, including site affinity, migratory connections, and movements, for appropriate national and trans-national management.

In the North Pacific, humpback whales feed across several feeding grounds, from California to Alaska and across to Russia^13^. Photo-ID and genotype matches (derived from analysis of skin biopsies) have shown that these whales migrate to at least four breeding grounds: the Western North Pacific including Philippines and Japan^24^, the Hawaiian Islands, coastal and offshore Mexico^25^, and the coast of Central America^13,26^. Hill et al.^27^ suggested a fifth breeding ground, possibly around the Mariana Islands, but this needs to be confirmed with more research in the region. Combined photo-ID and genetic data have led to the identification of these different populations in the North Pacific, which are designated as Distinct Population Segments (DPS) under the U.S. Endangered Species Act^2^. The Central America DPS is considered endangered; thus, it is important to understand migratory patterns in this region, especially given the current challenges in delimiting the Mexico and Central America populations that display some intertwined photographic recaptures^28^. Humpback whale site fidelity to breeding grounds^10,29–33^ and to specific feeding grounds^9,34,35^ is mainly determined maternally. The term “migratory herd”^44^ has been used to refer to a group of animals sharing the same foraging and wintering ground. Herds from more than one feeding area may be found in a single breeding ground. Current knowledge based on photo-identification shows the presence of two herds migrating toward California: the Mexican and the Central American herd^36^.

Humpback whales that migrate to the Central America breeding grounds have been studied with varying degrees of effort within the region, with research conducted off Guatemala^37–39^, El Salvador^40^, Nicaragua, Costa Rica and Panama^13,41^. Connectivity between the Central America breeding grounds and feeding grounds in various parts along the western coast of North America has been reported in the past. For example, mtDNA genetic analysis has inferred evidence of connectivity between coastal sites in Central America and Oregon-California^42^. Other studies have found migratory connections between Costa Rica/Panama and California, Oregon and Washington^26,41,43–46^. Humpback whales breeding in the Costa Rican and El Salvadorian waters appear to have a preference for central and southern California^40,45^. In contrast, population ecology and migration characteristics of humpback whales off the coast of Nicaragua have received less attention (see^47,48^).

The Central America humpback whale population was evaluated in 2004–2008 during a collaborative project in the North Pacific, entitled ‘Structure of Populations, Levels of Abundance and Status of Humpback Whales’ (SPLASH), as being a small population (500–600 individuals,^13^), but it was later identified to be slowly increasing during a follow-up study including southern Mexican whales in 2019–2021 (1496 individuals^22^). These numbers suggest some recovery of the population, although the annual growth rate is slow (1.6%;^22^) compared to that of other humpback whale (sub)populations feeding off the U.S. West Coast (8.2%), and breeding grounds such as Hawaii (6.5%) and Australia (9.6–10.5%)^13,49,50^. Collaborative efforts by various research groups in the North Pacific, and the development of a research collaboration and citizen science platform called “Happywhale” (happywhale.com) provide opportunities to obtain new information about migratory destinations, movement patterns and migratory timing observed in Nicaraguan waters. Happywhale uses fully automated fluke-matching software^51^, that can provide fluke matches from multiple times and locations.

The goal of this study was to determine the migratory destinations of humpback whales sighted off Nicaragua, considering site affinity and spatio-temporal patterns of movement. To address this objective, we combined 36 years of photographic recaptures available on Happywhale through international collaborations with a 10-year dataset collected at two sites in Nicaragua (“northern” and “southern”). Four specific questions were addressed in this study: (1) What are the migratory destinations of individuals breeding in Nicaragua? (2) Is there an influence of feeding and/or Nicaraguan breeding site of origin on the number of individuals observed at feeding sites? (3) What is the migratory timing of humpback whales that winter in Nicaragua? (4) Do individuals that breed in Nicaragua show site affinity for specific breeding and feeding sites?

## Results

### Individual identifications

The photo-ID catalogue from 2004 to 2008 and 2016 to 2021 (except 2019) of humpback whales identified off Nicaragua contained 176 individuals, identified by ventral fluke pattern, and included 167 non-calves and 9 calves. A total of 75 and 114 individuals were sighted, respectively, in northern and southern Nicaragua. A total of 13 individuals were sighted in both northern and southern Nicaragua (7.4%). Out of the 176 individuals, 148 individuals (84%) were matched with feeding areas.

### Migratory destinations

**Figure 1:**
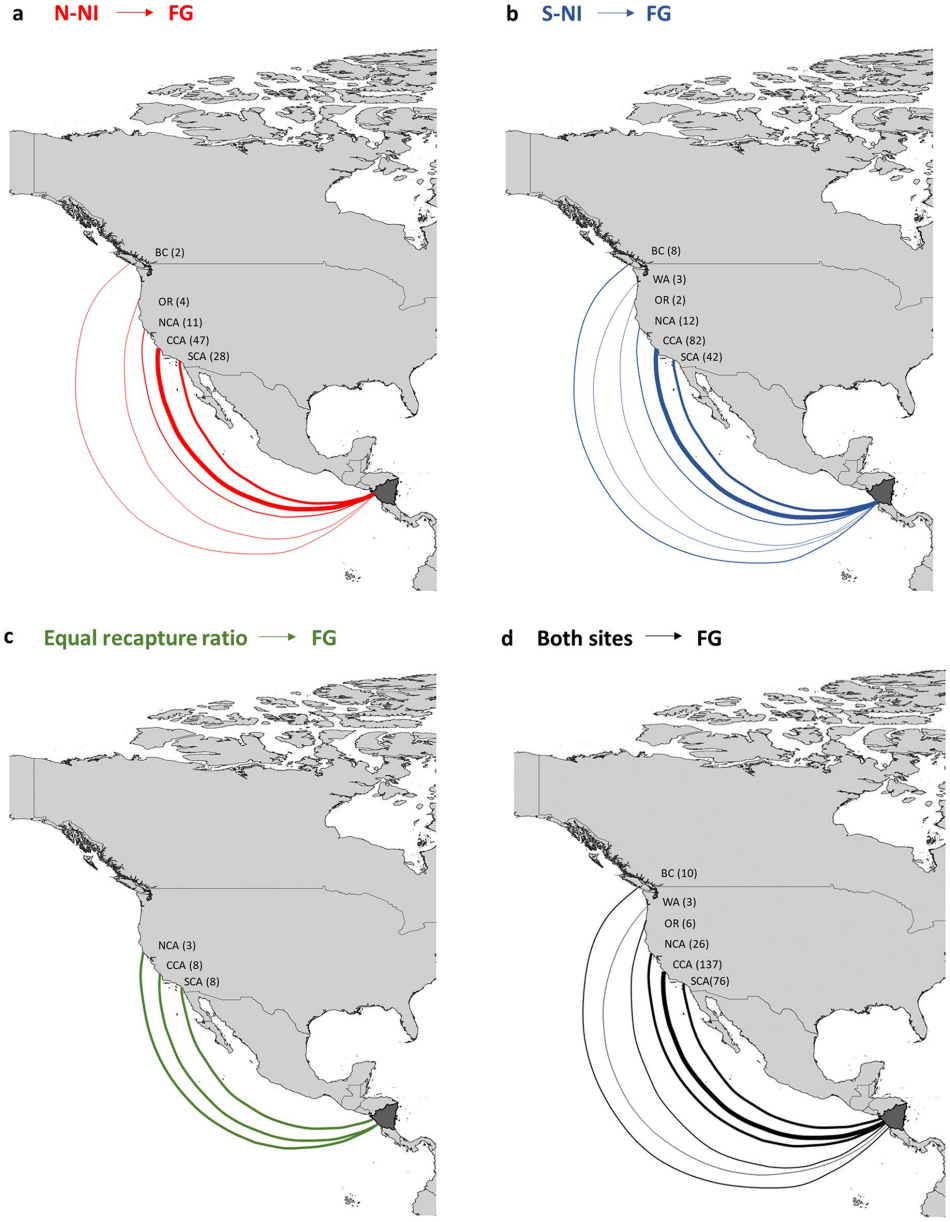
Migratory destinations of humpback whales observed off (**a**) northern Nicaragua (N-NI) (unique individuals* n* = 62), (**b**) southern Nicaragua (S-NI) (unique individuals* n* = 101), (**c**) individuals observed in both sites with equal recapture ratios (unique individuals* n* = 13) and (**d**) both the northern and southern sites combined. Numbers in brackets represent the number of individuals observed per feeding sites defined in the feeding ground (FG). Note that one individual could be observed in multiple feeding sites. Feeding sites: BC, British Columbia; WA, Washington; OR, Oregon; NCA, northern California; CCA, central California; SCA, southern California. The background map was created with the QGIS 3.22.5 software (www.qgis.org), using country administrative boundaries provided by Diva-GIS (http://www.diva-gis.org/).

Humpback whales from both northern and southern Nicaraguan sites were recaptured in feeding areas primarily along the U.S. West Coast and into British Columbia (Fig. 1a–d), except Washington in the case of whales sighted in northern Nicaragua (Fig. 1a). Individual humpback whales from both northern Nicaragua (N-NI) and southern Nicaragua (S-NI) were mainly sighted in central California (N-NI *n* = 47 and S-NI *n* = 82); southern California (N-NI *n* = 28 and S-NI *n* = 42); northern California (N-NI *n* = 11 and S-NI *n* = 12); Oregon (N-NI *n* = 4 and S-NI *n* = 2); Washington (N-NI *n* = 3); and British Columbia (N-NI *n* = 2 and S-NI *n* = 8) (Fig. 1a, b). A higher proportion (82%; *n* = 145) of whales from Nicaragua was recaptured in California; however, the number of individuals recaptured in California were mainly located in central (84%; *n* = 147) and southern California (44%; *n* = 76; note that some individuals were recaptured in multiple areas). Individual humpback whales from Nicaragua were found in all breeding sites, including mainland Mexico (*n* = 86; 49%), Baja California (*n* = 54; 31%), southern Costa Rica (*n* = 24; 14%), Guatemala (*n* = 23; 13%), El Salvador (*n* = 22; 13%), northern Costa Rica (*n* = 16; 9.1%), and Panama (*n* = 1; < 1%).

A linear model (LM) quantifying the influence of feeding site and/or Nicaraguan breeding site of origin on the number of individuals observed at these sites indicated that this was best explained by the additive effect between the Nicaraguan site and the corresponding feeding site (Supplementary Table S1; Model 1; AIC = 93.9). There were significant differences in the number of individuals among feeding sites (ANOVA; F = 10.2792; df = 5; p < 0.01) with more individuals observed in central California compared to all other feeding sites within this study. No significant difference in migratory destinations to feeding sites was found between northern and southern Nicaraguan sites based on the number of individuals (ANOVA; F = 1.2137; df = 1; p = 0.01).

### Migratory timing

**Figure 2:**
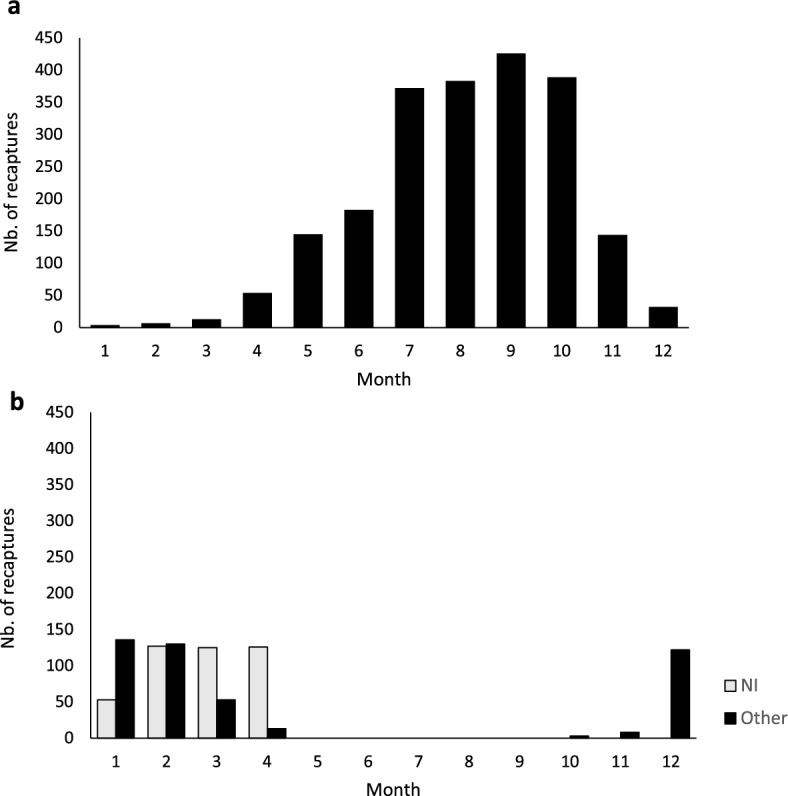
Migratory timing of humpback whales observed off the Pacific coast of Nicaragua with all recaptures pooled for (**a**) feeding grounds (*n* = 2073) and (**b**) breeding grounds (*n* = 897). NI, Nicaragua; Other, other breeding sites including Baja California; mainland Mexico; southern Mexico; Guatemala; El Salvador; northern Nicaragua; southern Nicaragua; northern Costa Rica; southern Costa Rica; Panama.

Whales from Nicaragua were recaptured in all months of the year in the feeding grounds. However, the number of recaptures increased in May and peaked in the July–October period (the main period identifications are obtained on feeding grounds), followed by a decrease in November (Fig. 2a). In the breeding grounds, humpback whales were observed between October and April, with peak recaptures in February (Fig. 2b).

**Figure 3:**
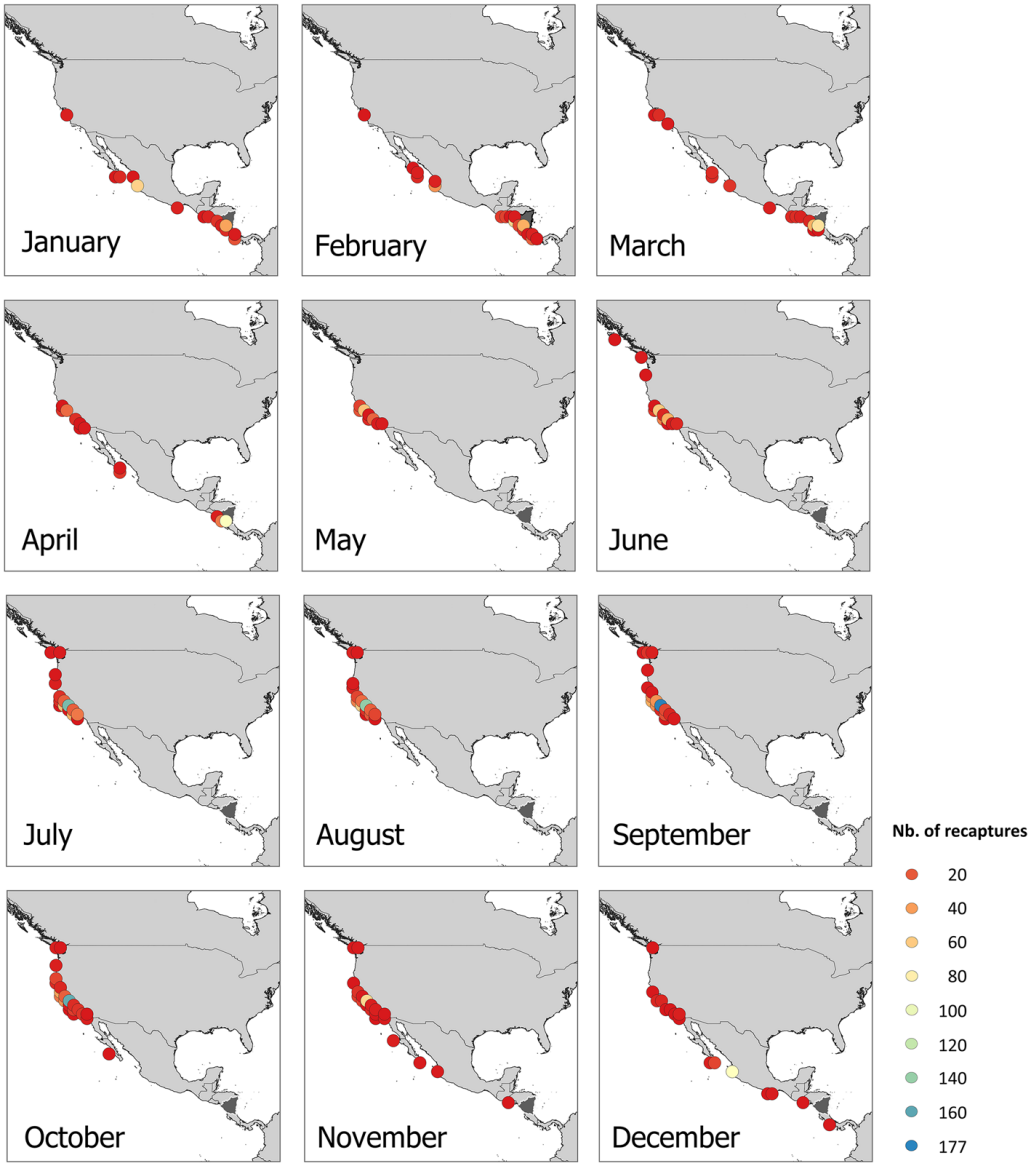
Spatio-temporal distribution of humpback whales observed off the Pacific coast of Nicaragua recaptured in feeding grounds (*n* = 2073) and in breeding grounds (*n* = 897). The background map was created with the QGIS 3.22.5 software (www.qgis.org), using country administrative boundaries provided by Diva-GIS (http://www.diva-gis.org/).

Some individuals began to migrate from feeding grounds to breeding grounds in October–November and arrived at Central American breeding ground as early as December (Fig. 3). Recaptures were obtained between January and April in both Mexico and Central America. In May, all recaptures occurred in California (U.S.) but, starting in June, a few Nicaraguan humpback whales were recaptured in more northerly feeding grounds, including Oregon and Washington in the U.S., and British Columbia in Canada (Fig. 3).

### Site affinity

**Figure 4:**
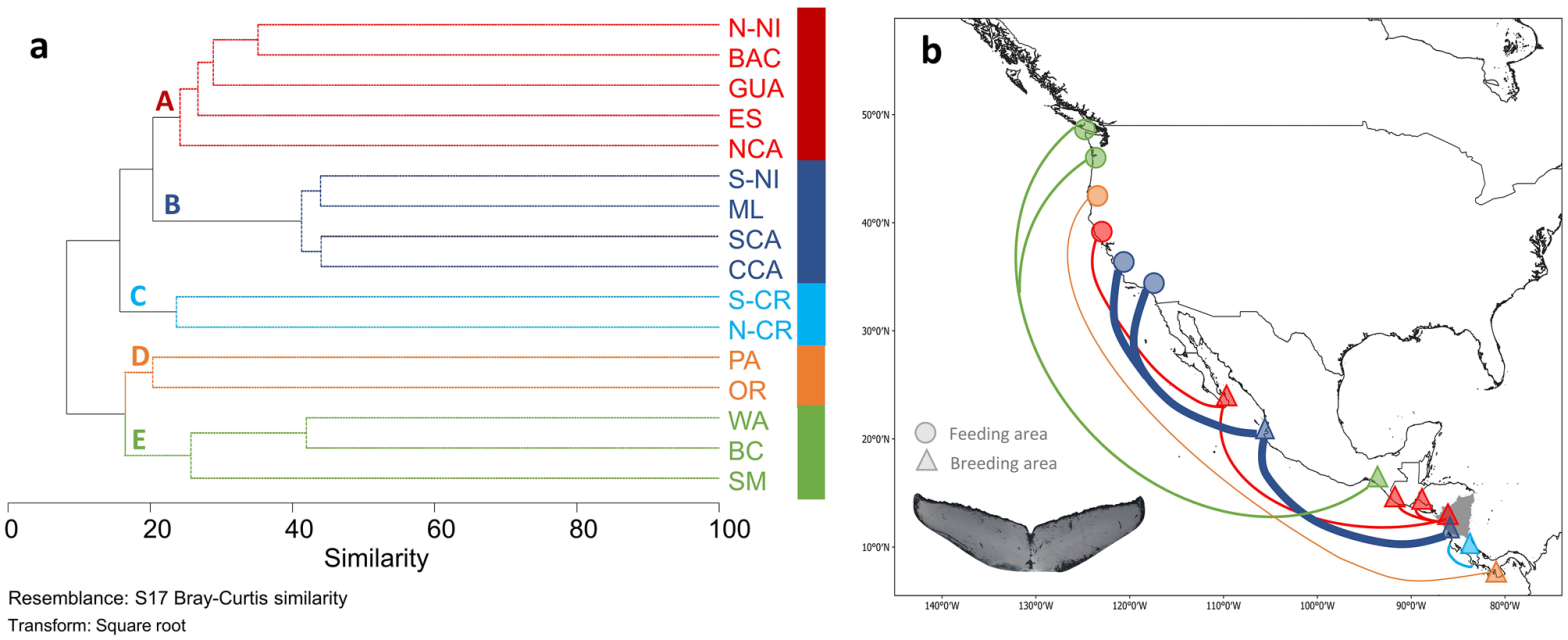
Site affinity and spatial structuring of humpback whale individual recaptures with a Nicaraguan site of origin. (**a**) Cluster analysis with the SIMPROF test based on similarity of individual recaptures. Identified clusters are indicated with a different letter with colored lines indicating a higher affinity between sites. The cluster was generated with PRIMER v. 7 software**.** (**b**) Representation of spatial structuring within the Nicaraguan whales based on individual photographic recaptures. Line thickness represents the level of similarity of each cluster based on the cluster analysis. BC, British Columbia; WA, Washington; OR, Oregon; NCA, northern California; CCA, central California; SCA, southern California, BAC, Baja California; ML, mainland Mexico; SM, southern Mexico; GUA, Guatemala; ES, El Salvador; N-NI = northern Nicaragua; S-NI, southern Nicaragua; N-CR, northern Costa Rica; S-CR, southern Costa Rica; PA, Panama. The background map was created with the QGIS 3.22.5 software (www.qgis.org), using country administrative boundaries provided by Diva-GIS (http://www.diva-gis.org/). The photograph was made available by Joëlle De Weerdt.

From a total of 176 individual identifications and 2971 recaptures of whales observed off Nicaragua, 431 recaptures were made in Nicaragua, 466 were made in other breeding grounds and 2074 in feeding grounds. The dendrogram showed that whales observed off Nicaragua connected with the following feeding sites: southern California, central California, northern California, Washington, Oregon, and British Columbia, and with all breeding sites considered in this study (*i.e.*, not including breeding grounds in Hawaii and Japan): mainland Mexico, Baja California, South Mexico, Guatemala, El Salvador, Costa Rica and Panama (Fig. 4). An exception was the Revillagigedo Islands (an oceanic island group off Mexico), for which no matches were found (Fig. 4).

A cophenetic correlation (R) of 0.83 indicated a strong positive correlation between the photographic recapture data and the resulting dendrogram (Fig. 4). The SIMPROF test indicated five significant different clusters based on site affinity (SIMPROF; R = 0.83; p < 0.05) (Fig. 4). Humpback whales sighted in Nicaragua were divided in five clusters based on site affinity (Fig. 4a). Cluster A (Fig. 4a) was represented by humpback whales sighted in the northern Nicaragua, Baja California, Guatemala, and El Salvador breeding sites and the northern California feeding site. Cluster B (Fig. 4a) was represented by humpback whales sighted in the southern Nicaragua and mainland Mexico breeding sites, and to southern and central California feeding sites. Cluster C had a higher affinity to northern and southern Costa Rica solely and did not display any affinity to other sites (Cluster C; Fig. 4a). Finally, other individuals had an affinity with southern Mexico and Panama breeding sites, and to the Oregon, Washington, British Columbia feeding sites (Clusters D and E; Fig. 4a). The shade plot with marginal dendrograms showed individual recaptures among clusters with a higher individual affinity in cluster B (southern Nicaragua, mainland Mexico, central and southern California (Fig. S3)).

## Discussion

Overall, humpback whales sighted off Nicaragua were recaptured all along the U.S. West Coast and British Columbia (Canada), as has been observed in previous research in other parts of Central America^13,26,43,45^. While low interchange between California and British Columbia/Oregon has been observed^52,53^, this study shows that a few individuals from Nicaragua were encountered in British Columbia (6%; *n* = 10)^54^, Washington (2%; *n* = 3) and Oregon (4%; *n* = 6). Even though survey effort is likely different among regions, our result of photographic recaptures for feeding and breeding grounds spans a long period (36 years), providing a first comprehensive examination of the migratory destinations of whales occurring off Nicaragua.

The recapture rates across feeding (Fig. 2a) and breeding grounds (Fig. 2b) of humpback whales sighted off Nicaragua coincide with known arrival and departure patterns of humpback whales in feeding (arriving in May and departing in November) and breeding grounds (arriving in December and departing in April)^55–57^, except with whales sighted off Guatemala, where they have been sighted as early as October and November^37^. An overall higher number of recaptures in feeding grounds compared to breeding grounds is likely related to the year-round presence of whale-watching boats in some areas and thus higher photographic effort, especially in Monterey Bay in central California. A high number of individual recaptures on one site can be due to site fidelity and longer seasonal residency time^9^. While differences in the intensity of the search effort in breeding and feeding grounds represent a source of potential bias, our results provide valuable information on spatio-temporal movement patterns of whales observed in Nicaragua. Humpback whales were sometimes observed in feeding grounds during the winter/spring (January to May), presumably because: (1) they cannot find enough food to support the onset of their migration^10^; (2) some females and juveniles are not migrating annually^58,59^; (3) individuals can present short seasonal residency times in breeding grounds (*e.g.,* staying only 2–4 weeks in breeding grounds)^60–62^; and/or (4) pregnant females remain longer on feeding grounds compared to other groups^5^. The small number of recaptures of Nicaraguan whales in breeding grounds during May and June is likely due to low whale densities in these months in coastal areas and/or to the lack of surveys during these months and/or to the fact that whales may migrate to feeding grounds along an offshore route (i.e., beyond the reach of our near-shore observation area). The decrease in recaptures in feeding grounds during November, and the concomitant increase in recaptures in breeding grounds from November onwards indicate that humpback whales start their migration to Nicaragua during this time of the year (Fig. 2a, b). Multiple recaptures of humpback whales observed off Nicaragua were made in Baja California and mainland Mexico. It has been previously suggested that both sites represent a migratory corridor for whales migrating from feeding grounds to Nicaragua^28,43^.

Previous photo-ID studies have shown broad-scale site fidelity at the scale of countries, for example the individuals from the Southeastern Pacific population sighted off Costa Rica and Panama, which are recognized by the International Whaling Commission as part of Breeding Stock G^63^. In Alaska, individual fine-scale site fidelity was found within a population between two locations located approximately 550 km apart^9^. The same was observed between Madagascar and Mayotte in the Southwestern Indian Ocean, habitats located 300 km apart^64^. Humpback whales can travel between 17 and 123 km per day on average^15–20^, allowing those individuals wintering off Nicaragua to easily swim the 250 km that separates the two study sites (northern and southern Nicaragua) in just a few days. At the same time, the very few recaptures between coastal waters of northern and southern Nicaragua study sites^47^ and the differences in clustering for both study sites could suggest the existence of spatial structuring within Nicaragua though this may also be the result of the difference in timing of the identifications from these two areas with many of the northern Nicaragua identifications coming from 2004 to 2008. The occurrence of apparent spatial structuring provides insight into the relationship between the Central America and mainland Mexico humpback whale DPSs, the boundaries of which have been difficult to determine^28^. The fact that some Central America DPS whales migrate along the mainland Mexico coastline to reach their breeding ground complicates making clear delineations between them. This is worth considering in the definition of two or more population units as suggested in previous studies^28^.

Our results showed that a higher number of individuals sighted off Nicaragua were found in central California independently of the site they originated from. While the results of our linear model showed that a higher number of individuals were sighted in central California, the cluster analysis based on photographic recapture rates indicated that individual site affinity was higher within central California for southern Nicaraguan whales specifically. Central California has a much larger number of identifications of humpback whales than other regions which was at least one factor in the high number of matches to this region. Northern Nicaraguan humpback whales can be found in central California, however, based on photographic recaptures and site affinity, these individuals preferred the northern California feeding site. We are not excluding that this was the result of potential temporal structuring due to annual differences in sampling effort between northern and southern Nicaragua. Spatial structuring can be based on maternally inherited factors or on social needs of the population (*e.g.,* calving, breeding, occasional feeding)^30^. In Central America, Guatemala appears to represent a migratory transit zone based on residency time^38^, whereas northern Nicaragua appears to be a calving and resting area based on social group presence and behaviours^47^. Waters off southern Nicaragua could be a destination for adults involved in breeding activities, while at the same time taking advantage of potential incidental feeding, which has occasionally been observed in the region^48^. Southern Costa Rica likely serves as a nursing area due to its calm and warm waters^41,43,65^. The fact that whales seen in Nicaragua were recaptured across different clusters including southern Mexico, northern and southern Costa Rica and Panama might indicate that humpbacks were transiting through Nicaragua toward these breeding sites. Alternatively, this could potentially reflect exploratory behaviors by males searching for receptive females^17^. Even if waters along the Mexican coast were suggested to be a migratory corridor, it could be either, or both, a breeding ground or serve as a non-breeding stopover for humpback whales migrating toward Central America^28,66^. Further research effort at a regional scale is needed to understand how this spatial structuring has emerged and how it may be related to habitat use and/or to temporal variations. This research effort should include genetic sampling, additional photo-identification sampling along all Central American countries and Mexico (including additional Southern Mexican databases), as well as data on behavior and group composition for refined interpretations. Our findings on the feeding ground destinations of whales sighted off Nicaragua aligns with the notion of a single humpback whale herd that migrates between Central America and the U.S. West Coast and Southern British Columbia^36^. However, how this migratory herd uses Pacific Mexico remains highly debated^28,67^. Our study found recaptures between whales sighted off Nicaragua and three locations off the Pacific coast of Mexico. Additional research into migratory connections and divisions between the Central America and Mexico DPS will aid in further eluding the exact identity and habitat use (e.g., migratory, mating and/or calving) of the herds^36^ that utilize these two Eastern North Pacific breeding grounds. Our research provides additional insight into the complexity of DPS classifications and shows that the Central America and Mexico DPSs might be more intertwined than previously thought. Further research should include this information to provide a critical analysis of management actions at national and international levels, since DPS assignment can affect conservation planning.

### Potential biases

The research effort from the Happywhale database is spatially and temporally heterogeneous, sourced from combined scientific and opportunistic data that is not normalized for effort. Data from mainland Mexico, Southern Mexico and Central America breeding areas are > 90% research sourced (except Baja California (research (34.4%) and opportunistically (65.6%) sourced), while data from feeding areas are heterogenous in source (both research (34.6%) and opportunistically (65.4%) sourced)^68^. This heterogeneity may bias results if the relatively small dataset of Nicaraguan whales contains whales that migrate to feeding and /or breeding areas with less sampling effort; therefore caution should be taken when comparing this study with other research efforts^68^. Further, heterogeneity in effort outside of the peak breeding season along Central American countries can introduce potential biases in both migratory timing, resulting in less or more detections in certain months (*e.g.,* absence or limited monitoring in December).

## Conclusion

This study reports for the first time detailed migratory timing, destinations and site affinity of humpback whales wintering in waters off Nicaragua. The results of the photographic recaptures suggest the presence of possible spatial or temporal structuring between different breeding and feeding grounds of humpback whales seen off Nicaragua. Identifying spatial structuring has important implications for better understanding humpback whale population ecology. We suggest extending this analysis to all Central America whales to gain a better understanding of the underlying explanation for spatial structuring within the Central America and Mexico, which would help clarify the differences between both populations. If further fine-scale spatial structuring is confirmed through the regional-scale analysis, this could imply that conservation actions at the national level are crucial for the conservation of humpback whales from Central America. This study demonstrates that collaborative photo-identification efforts between scientists and to some extent citizen scientists, can be applied to assess migratory timing and destinations, and site fidelity.

## Methods

### Photo-identification and study areas

Photo-ID allows to identify individual whales, using the method developed by Katona et al.^7^. This method uses the natural marks and coloration patterns on the ventral side of photographed flukes as well as the shape, size and scarring of both the left and right sides of the dorsal fin^69^. Dedicated boat-based surveys were conducted during calm to moderate sea states (i.e., Beaufort Scale 0–4) on a 6-m fiberglass boat with a 75-hp motor, at two study sites off the Pacific coast of Nicaragua, Central America. The first location, Padre Ramos, 12°45′ N–87°29′ W (northern Nicaragua; hereafter N-NI), was surveyed by Cascadia Research Collective (CRC) during the boreal winters (surveys from late January to early March) of 2004–2008, and by Association ELI-S (ELI-S) during the winter breeding seasons of 2016–2018. The second location, San Juan del Sur, 11°15′ N–85°52′ W (southern Nicaragua; hereafter S-NI) was surveyed by ELI-S during the breeding seasons between 2016 and 2021 (except 2019) (Figs. S1, S2). Boat-based surveys by ELI-S were dedicated to cetaceans and were conducted ad libitum*,* while surveys by CRC were ad libitum as well, but dedicated to humpback whales and used acoustics as a complementary tool for whale detection. A total of 12,426 km was surveyed in northern Nicaragua and 8149 km in southern Nicaragua.

When a humpback whale (or group; *i.e.,* animals within two body lengths coordinating swimming and diving behaviours for at least one surfacing^34^) was encountered, GPS position, time and photographs were taken with a D7100 DSL Nikon camera and a 55–300 mm lens. Photographs from opportunistic sightings in 2014 and 2019 (*n* = 3) contributed by fishermen and tourists were added to the ELI-S database. A comprehensive photo-identification catalog was built based upon identifications of individual humpback whales by CRC and ELI-S. To be included in our catalog, photographs of sufficient quality were screened based on four parameters: contrast (range of tones in the image), angle (angle of the dorsal fin or fluke to the camera), partial (visible proportion of the fin) and clarity (sharpness of the image). Flukes of calves were kept in the analysis since fluke coloration patterns might be recognized in case of short-term recapture^70^. The Nicaragua photo-ID catalog from the period 2016–2020 was uploaded to the platform Happywhale^51^. Data, including location, date, time and photographs, collected between 1986 and 2021 sourced from a North Pacific Ocean-wide collaboration^68^ were downloaded from Happywhale. A recapture was defined as the photographic recapture of an individual whale. Recaptures within breeding or feeding grounds were assessed through comparisons of all available datasets on the Happywhale platform. Dorsal fins were used in the ELI-S database to infer recaptures within the Nicaraguan breeding site.

Feeding grounds off the western coast of North America were divided, based on Canadian and U.S. state limits and equidistant separations within larger states, into the following six areas (from south to north): (1) Southern California (SCA), extending between the longitudes 32°42′ N and 34°57′ N; (2) Central California (CCA), extending between the longitudes 34°57′ N and 38°26′ N; (3) Northern California (NCA), extending between the longitude 38°26′ N and the border with Oregon (42°01′ N); (4) Oregon State (OR); (5) Washington State (WA); and (6) southern British Columbia, Canada (BC) (Fig. S1). The Mexican and Central American breeding grounds were divided according to researchers’ study areas and were defined as follows (from south to north): Panama (PA); Costa Rica south (S-CR); Costa Rica north (N-CR); Nicaragua south (S-NI); Nicaragua north (N-NI): El Salvador (ES); Guatemala (GUA); Southern Mexico (SM); mainland Mexico, including Jalisco and Nayarit states (ML); and Baja California Peninsula (BAC) (Fig. S1).

### Migratory destinations

In Nicaragua, individuals recaptured at only one site, independently of the month and the season, were assigned to one Nicaraguan area (N-NI or S-NI). Whenever an individual was recaptured at both sites, recapture ratios were evaluated but only one recapture per individual per day was considered. If a biased recapture ratio was observed (A:B, with A > B; *e.g.,* 2:1, 3:1) towards one site, the whale was assigned to the site with the highest ratio. If an equal recapture ratio was observed (*e.g.,* 1:1; 2:2), whales were not assigned to a particular site and were analyzed separately in their migratory destination. An individual observed in a feeding ground was assigned a “1” when sighted and a “0” when not reported, independently of the season, the year and/or the number of recaptures. The sum of the individuals sighted for each feeding ground was used to infer the migratory destinations of whales observed at the two sites in Nicaragua. Note that one individual can be counted on more than one feeding site. Migratory destinations were assessed for both sites (including equal recapture ratios), for each site and equal recapture ratios.

To examine whether individual counts are explained by the feeding site they were recaptured and/or the Nicaraguan site they originate from, linear models (LM) were applied in the statistical program R to test the differences in individual counts (Ind; based on 0–1 classification). A feeding site could have a maximum count of 176 individuals (total number of individuals in this study). Predictor variables included feeding grounds (FG) with six different regions including BC, WA, OR, NCA, CCA, SCA; and Nicaraguan sites (Sites), with two levels including N-NI and S-NI. The response variable was the number of unique individuals. The LM allowed to assess differences between levels of each predictor variables on the response variables. Model selection was based on Akaike’s Information Criterion (AIC) minimization. Model assumptions including homogeneity and normality of residuals were verified the Shapiro test and the *ncvTest* function of the *car* package in R^71^. If the homogeneity of variance of the model was significant, we applied *white.adjust* to test whether heteroscedasticity impacted the model. A post-hoc test was applied with the *multcomp* package to analyze the amount of variance that contributed to the individuals recapture based on the different levels of the predictor variable^72^.

### Migratory timing

To estimate the migratory timing of humpback whales observed off Nicaragua, photographic recaptures of individuals were pooled per month for each previously defined breeding and feeding ground. Migratory timing is defined as the spatial and temporal location of recaptures, which allows to understand where and when humpback whales observed off Nicaragua are located. Monthly recaptures were plotted on QGIS 3.22.5^73^ using the *countpoint in polygon* and the *centroid* tool. Photographic recaptures were pooled per month and mapped accordingly.

### Site affinity

Based on their geographic coordinates, recaptures were classified independently of the season into the corresponding feeding and breeding sites defined in this study. To test differences in individual site affinity along the whales’ migratory route, a cluster analysis was conducted based on the number of recaptures for each individual. The Bray–Curtis dissimilarity index allowed the calculation of individual recapture dissimilarities between sites. The dissimilarity index is defined according to following formula^74^:

$${\updelta }_{{{\text{jk}}}} = 100 - \frac{{\sum\nolimits_{{\text{i = 1}}}^{{\text{p}}} {\left| {{\text{y}}_{{{\text{ij}}}} - {\text{y}}_{{{\text{ik}}}} } \right|} }}{{\sum\nolimits_{{\text{i = 1}}}^{{\text{p}}} {\left| {{\text{y}}_{{{\text{ij}}}} {\text{ + y}}_{{{\text{ik}}}} } \right|} }}$$

where y_ij_ = score (count) for ith individual in jth sample. With δ = 0 being no dissimilarity; δ = 100 total dissimilarity.

Clusters were constructed from the Bray–Curtis dissimilarity matrix, using the group-average linkage method to build a dendrogram to display grouping among similar sites (high number of recaptures). A square-root transformation was applied to counterbalance the similarities^75^ between sites with a high and low number of recaptures, thus reducing the effect of bias in the sampling effort. The cophenetic correlation allowed the examination of the degree of representation of the similarity matrix and the dendrogram. This was a Pearson matrix correlation between the similarity matrix and the distance matrix through the dendrogram between the corresponding pairs of samples. Values for the cophenetic correlation close to 1 indicated a good representation of similarity. To test for significance among groups the Similarity Profile Analysis (SIMPROF) at alpha = 0.05 was used. Shade plots were generated to assess relationships between clusters and individual recaptures. Clusters, SIMPROF and shade plots were implemented using the PRIMER v. 7 software^76^.

### Supplementary Information

Supplementary Information.

## Data Availability

The datasets from the citizen science platform are available from Happywhale platform on request, contact Ted Cheeseman (ted@happywhale.com) for Happywhale data and Joëlle De Weerdt (corresponding author) for data in Nicaragua.
