# Supplementary material for: Migratory destinations and spatial structuring of humpback whales (Megaptera novaeangliae) wintering off Nicaragua
Source: Sci Rep. 2023 Sep 13;13:15180. doi: 10.1038/s41598-023-41923-7 (PMC10500005; doi:10.1038/s41598-023-41923-7)
Supplement: Supplementary file 1 — Supplementary Information. [file 41598_2023_41923_MOESM1_ESM.pdf]

Migratory destinations and spatial structuring of humpback whales (*Megaptera novaeangliae*) wintering off Nicaragua

Joëlle De Weerdt[1,2,*], Aldo S. Pacheco[3], John Calambokidis[4], Melvin Castaneda[5], Ted Cheeseman[6], Astrid Frisch-Jordán[7], Frank Garita Alpízar[4], Craig Hayslip[8,9], Pamela Martínez-Loustalot[10], Daniel M. Palacios[8,9], Ester Quintana-Rizzo[11], Nicola Ransome[5,12], Jorge Urbán Ramírez.[9], Phillip Clapham[13], Tom Van der Stocken[2]

[1]Association ELI-S, Education, Liberté, Indépendance - Scientifique, Allée de Verdalle 39, 33470 Gujan-Mestras, France. www.eli-s.com
[2] Biology Department, Vrije Universiteit Brussel, VUB, Pleinlaan, 1050 Brussel, Belgium
[3] Facultad de Ciencias Biológicas, Universidad Nacional Mayor de San Marcos, Av. Carlos Germán Amezaga #375, Lima, Perú
[4] Cascadia Research Collective, 218½ W 4th Avenue, Olympia, Washington 98501, USA
[5] Fundación Naturaleza El Salvador
[6] Happywhale.com, Marine Ecological Research Centre, Southern Cross University, Lismore, NSW, Australia
[7] Ecología y Conservación de Ballenas, A.C. ECOBAC, México
[8] Department of Fisheries, Wildlife, and Conservation Sciences, Oregon State University, Newport, Oregon, USA
[9] Marine Mammal Institute, Oregon State University, Newport, Oregon, USA
[10] Departamento de Ciencias Marinas y Costeras, Universidad Autónoma de Baja California Sur, La Paz, México
[11] Emmanuel College | Simmons University, USA
[12] Murdoch University (Harry Butler Institute), Perth, Western Australia, Australia
[13] Seastar Scientific, 27605 Hake Rd SW, Vashon, WA 98070, USA

Corresponding author: [*]eliscientific@gmail.com

Supplemental Material

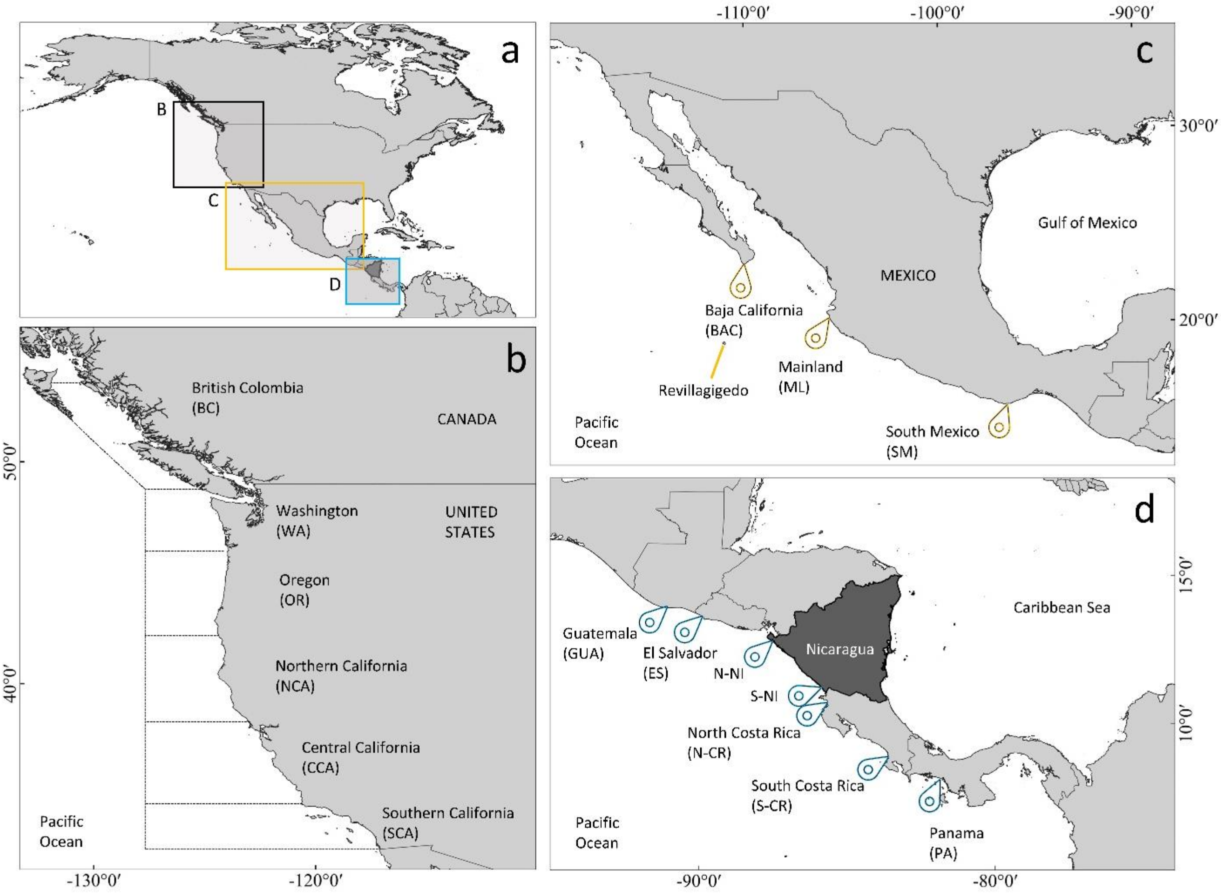

Supplementary Figure S1. Location of study areas in the eastern North Pacific (a) where photo-identification of humpback whales (*Megaptera novaeangliae*) was conducted through both scientific (b, c, d) and citizen science programs (b). Feeding grounds are depicted in (b) and breeding grounds in (c, d). The background map was created with the QGIS 3.22.5 software (www.qgis.org), using country administrative boundaries provided by Diva-GIS (http://www.diva-gis.org/).

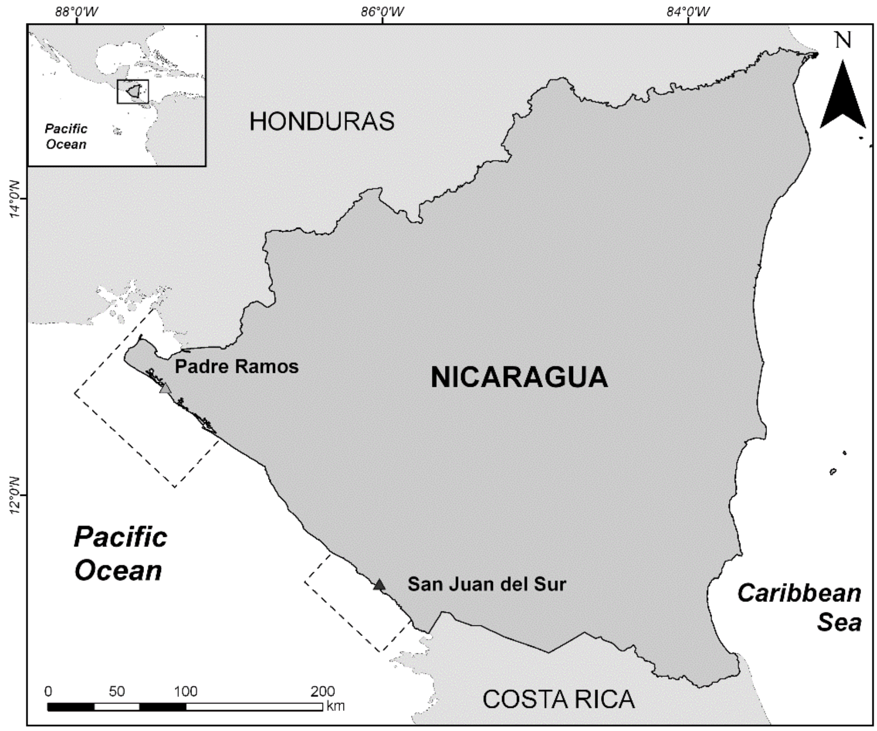

Supplementary Figure S2. Study area covered in northern study area (N-NI) and southern study area (S-NI) in Nicaragua (From De Weerdt *et al.* 2022). The background map was created with the QGIS 3.22.5 software (www.qgis.org), using country administrative boundaries provided by Diva-GIS (http://www.diva-gis.org/).

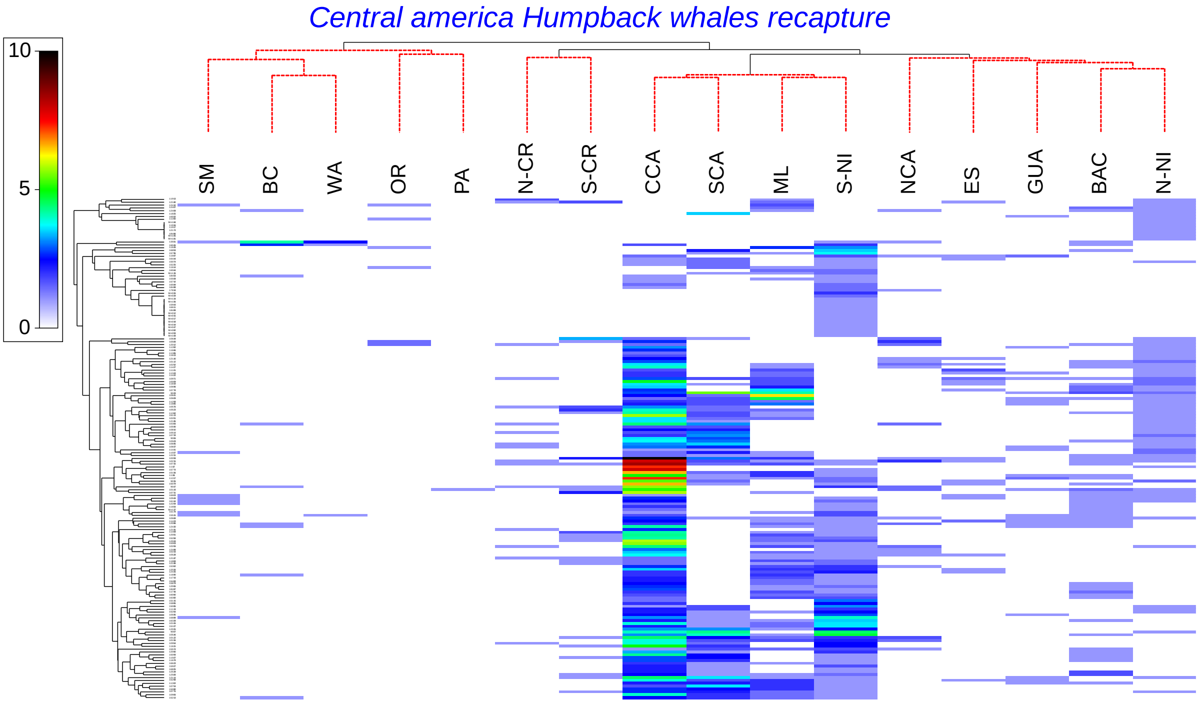

Supplementary Figure S3. Shade plot with marginal dendrograms for individuals (left axis) and study sites (top axis) showing global relationships among clusters of individual recaptures. Every line represents an individual and color scale represents the number of recaptures in an area (n = 2970). BC= British Columbia, WA= Washington; OR= Oregon; NCA= northern California; CCA = central California; SCA = southern California, BAC = Baja California; ML = Mainland Mexico; SM= Southern Mexico; GUA = Guatemala; ES = El Salvador;

N-NI = northern Nicaragua; S-NI = southern Nicaragua; N-CR = northern Costa Rica; S-CR = southern Costa Rica; PA= Panama. The shade plot was generated with PRIMER v. 7 software.

Supplementary Table S1. Linear model selection results based on minimization of Akaike's information criterion (AIC) for the number of individuals recaptured in function of the Nicaraguan study site (N-NI versus S-NI) and Feeding ground (FG). † Selected model (Model 1).

**Model**	**Variables**	**df (among)**	**df(within)**	**R^2**	**F**	**p**	**AIC**	**ΔAIC**
0	Ind ~ 1 (Null Model)	1	11	0.00	-	-	114.5	-20.6
1†	Ind ~ Site + FG	1	11	0.93	11.72	**0.01**	93.9	0.0
2	Ind ~ Site	1	11	0.04	0.40	0.54	116.0	-22.1
4	Ind ~ FG	1	11	0.02	10.23	0.01	97.4	-3.5
